# Urine carcinoembryonic antigen levels are more useful than serum levels for early detection of Bilharzial and non-Bilharzial urinary bladder carcinoma: Observations of 43 Egyptian cases

**DOI:** 10.1186/1477-7819-5-4

**Published:** 2007-01-15

**Authors:** Gamal M Saied, Wafaa H El-Metenawy, Mohamed S Elwan, Nazar R Dessouki

**Affiliations:** 1 Department of General Surgery, Faculty of medicine, Cairo University, Cairo, Egypt; 2 Department of Radiation Oncology, Faculty of Medicine, Cairo University, Cairo, Egypt; 3 Department of General Surgery, St. Bernard's Hospital, Gibraltar, UK

## Abstract

**Background:**

Both urinary bilharziasis and urothelial neoplasia are associated with increased production of tissue carcinoembryonic antigen (CEA).

**Patients and methods:**

Urine and serum CEA were determined in 43 patients with urinary bladder carcinoma including 22 post bilharzial and 21 nonbiharzial cases, in addition to 10 normal control cases.

**Results:**

A significant increase was detected in both urine and serum CEA levels with bladder carcinoma compared to control cases. Urinary CEA was significantly elevated in 86% of bilharzial, versus 62% in nonbilharzial bladder carcinoma. Only 10.5% of control cases had urinary CEA elevation. The mean urinary CEA in bilharzial, was higher than that of nonbilharzial carcinoma, but the difference was not statistically significant. There was a definite relationship between urine CEA and the stage of malignancy; the higher the stage, the higher the level of urine CEA. No relationship could be detected between the stage of malignancy and serum CEA, or between the grades of malignancy and urine or serum CEA levels.

**Conclusion:**

Urinary CEA is more useful than serum CEA in the early detection of urotherlial carcinoma particularly if provoked by bilharziasis. Its level is also correlated with the tumor stage.

## Background

Carcinoembryonic antigen (CEA) a specific product of neoplasia derived from the endoderm and is supposed to have a potential value in screening, diagnosis and follow-up of patients suspected of having urothelial carcinoma [1]. It has also been detected in other types of normal human tissue including prostate, uterus and spleen. This aroused fears of false positive results and of compromising specificity if further enhancement of sensitivity was tried [2]. Urinary CEA measurement and cytological examination are two noninvasive procedures that were compared and found to yield similar frequencies of positivity. Simultaneous performance of these two tests increased the yield of positive results to 86% [3]. It was also suggested that assay of urinary CEA might provide an alternative to urinary cytology for industrial screening of high-risk population. In hospital practice, it was thought to be useful alongside cytology and cystoscopy in primary diagnosis and routine follow-up of patients with urothelial tumors after treatment to detect early recurrences. It was also found to add to the information of T classification [4,5]. The finding that T1 and *in situ *carcinoma can yield raised values of CEA is of potential importance as it is this group of tumors which is difficult to detect by urinary exfoliative cytology [6].

Urinary CEA was first suggested to be particularly helpful to evaluate urothelial dysplasia [7] and in patients with bilharzial chronic cystitis it may affords a valuable screening test for premalignant lesions and malignant transformation [8,9].

Raised serum CEA was recorded in association with invasive tumors or the presence of metastatic disease [10,11]. With regards CEA tissue level, it was found to be higher in malignant vesical urothelium than in the control group, concentrations were much higher with infiltrating tumors [12]. Normalization of CEA level in follow-up of treated cases points to successful management [13]. On the other hand, local recurrence or multiple metastases were found to be associated with elevated CEA [14].

Bilharzial carcinoma of the urinary bladder was found to represent a distinct clinico-pathological entity different from nonbilharzial carcinoma [15]. The aim of this study is to throw light on the value of urinary and serum CEA in the diagnosis of carcinoma of the urinary bladder and to see if there is any difference between bilharzial and nonbilharzial carcinoma as regards production of CEA, a new point not handled before.

## Patients and methods

This study was conducted at Kasr EI-Aini University Hospitals, Cairo/Egypt from April 2002 through April 2005. Forty three patients having proven carcinoma of the urinary bladder beside 10 control cases were enrolled in the study and categorized into three groups. Group I included 22 patients having bilharzial carcinoma; group II included 21 patients having nonbilharzial carcinoma while group III consisted of 10 normal controls with no infection or malignancy of the urinary tract or malignancy elsewhere. Radical cystectomy was done to patients of the first two groups. Transuretheral resection was not attempted even in early lesions due to the prevalence of multicentricity in bilharzial cases and to standardize the treatment procedure.

For every case, urine and serum CEA were measured. Patients showing evidence of acute urinary tract infection were excluded. Samples of 10 ml of midstream morning urine were collected. Samples with 5 pus cells or more/HPF were discarded. Five ml of blood were obtained from fasting individuals after at least 6 hours of stopping smoking. No additives or preservatives were necessary to maintain the integrity of the specimens. Grossly hemolysed samples were discarded. CEA was measured in both urine and serum by monoclonal enzyme immunoassay (EIA) method using a commercially available kit from Abbott. The normal adult range by this method is less than 3 ng/ml in serum and less than 30 ng/ml in urine. Insignificant minimal elevations are found in heavy smokers.

For patients with carcinoma of the urinary bladder, the following was done: a detailed clinical historywas taken and patients with a positive history of bilharziasis were excluded from group II, but this was not sufficient to put them in group I. Secondary bacterial infections were excluded before enrollment as it may affect urinary CEA values. No evidence that bilharziasis alone (in the absence of frank premalignant lesions or cellular dysplasia) has such effect [8]. Clinico-pathological examination included bimanual examination to assess operability particularly in bilharzial carcinoma where the mobility (not the size) is important. Staging was done according to the TNM system of UICC [16] for non-bilharzial cases it was done by CT scans and confirmed postoperatively on the resected specimens. Cystoscopic examination and biopsy were performed. Radical cystectomy was done for all cases and the specimens were examined for type and grade of malignancy, pathological staging and the presence or absence of associated bilharzial cystitis.

## Results

There was a definite relationship between the stage of malignancy and the level of urine CEA, the higher the stage the higher the level of urine CEA. There was no relationship between the stage of malignancy and the level of serum CEA or between the grade of malignancy and the levels of both urine and serum CEA. The evidence was observed using correlation coefficient (r). Table 1 shows the relation of urine and serum CEA levels with type, grade and stage in bilharzial carcinoma. The relation is direct with correlation coefficient 0.131 at *p *= 0.0236. Table 2 shows same relation in non-bilharzial cases (correlation coefficient is -0.25 at *p *= 0.0007). In control cases correlation coefficient was 0.60 at *p *= 0.065. Finally Table 3 compares the percentage of cases with high levels of urine and serum CEA in the three groups. Table 4 show percentage of cases having urine CEA above 10 ng/ml and serum CEA above 5 ng/ml.

**Table 1:** CEA in urine and serum, histopathological type, grade of malignancy and pathological stage of bilharzial carcinoma of the urinary bladder.

Case no	Urine CEA	Serum CEA	Histological type	Grade of malignancy	Pathological staging
1	55	5	Squamous cell	II	P2
2	47	-	Squamous cell	I	P2
3	9	17	Squamus cell	I	P1
4	72	-	Squamous cell	I	P2
5	40	7	Squamous cell	II	P2
6	140	4	Squamous cell	I	P3
7	114	7	Squamous cell	III	P3
8	33	17	Squamous cell	II	P2
9	10	-	Squamous cell	I	P2
10	13	-	Squamous cell	I	P2
11	4	35	Squamous cell	I	P1
12	14	8	Squamous cell	II	P2
13	85	0	Transitional cell	III	P2
14	105	0	Squamous cell	II	P3
15	100	5	Transitional cell	II	P2
16	70	1	Squamous cell	I	P3
17	28	0	Squamous cell	II	P2
18	270	9	Adenocarcinoma	III	P3
19	135	35	Squamous cell (verrucous type)	I	P3
20	20	0	Squamous cell	III	P2
21	105	8	Squamous cell	II	P2
22	165	0	Transitional cell	II	P3

Mean urine CEA in bilharzial carcinoma: 75.09 mg/ml (Standard deviation: 64.26 ng/ml)

Mean serum CEA in bilharizal carcinoma = 5.03 ng/ml (Standard deviation = 5.41 ng/ml)

**Table 2:** CEA in urine and serum, histopathological type, grade of malignancy and pathological stage of nonbilharzial carcinoma of the urinary bladder.

**Case No**	**Urine CEA (ng/ml)**	**Serum CEA (ng/ml)**	**Histological Type**	**Grade**	**Stage**
1	22	8	Transitional	III	P3
2	10	10	Transitional	III	P1
3	14	--	Transitional	III	P2
4	30	--	Transitional	III	P3
5	8	4	Anaplastic	IV	P2
6	26	3.5	Transitional	III	P2
7	160	2.5	Transitional	III	P3
8	17	3	Transitional	III	P1
9	13	3	Transitional	III	P2
10	3	5	Transitional	III	P1
11	165	0.5	Transitional	III	P2
12	135	0.5	Anaplastic	IV	P3
13	15	0.5	Transitional	II	P2
14	4	0.5	Transitional	II	P2
15	120	1	Transitional	III	P3
16	75	3	Transitional	II	P1
17	3	1	Transitional	II	P1
18	5	0	Squamous	III	P3
19	5	3	Transitional	III	P2
20	1	0	Transitional	III	P2
21	90	3	Squamous	II	P1

Mean for urine CEA in non-bilharzial carcinoma = 43.86 ng/ml. (Standard deviation = 55.70 ng/ml)

Mean for serum CEA in non-bilharzial carcinoma = 2.74 ng/ml. (Standard deviation = 2.68 ng/ml)

Mean urine CEA in squamous cell carcinoma = 58.26 ng/ml. (Standard deviation = 42.12 ng/ml)

Mean urine CEA in transitional cell carcinoma = 50.14 ng/ml. (Standard deviation = 58.31 ng/ml)

Mean serum CEA in squamous cell carcinoma = 5.60 ng/ml. (Standard deviation = 5.41 ng/ml)

Mean serum CEA in transitional cell carcinoma = 2.37 ng/ml. (Standard deviation = 2.79 ng/ml

**Table 3:** CEA in urine and serum of the control cases.

Case No.	Urine CEA (ng/ml)	Serum CEA (ng/ml)
1	1	2
2	0	2
3	1	1.5
4	0	2
5	0.5	2.5
6	1.5	1.5
7	0	1
8	2	0.5
9	2	0.5
10	1	0.5

Mean for urine CEA in of control cases = 0.9 ng/ml (Standard deviation = 0.77 ng/ml)

Mean for serum CEA of control cases = 1.4 ng/ml (Standard deviation = 0.74 ng/ml)

**Table 4:** Percentage of cases having urine CEA above 10 ng/ml and serum CEA above 5 ng/ml.

**Group**	**Urine CEA**	**Serum CEA**
I	86%	39%
II	62%	10.5%
III	0%	0%

## Discussion

Since CEA is present in the normal urothelium, destruction and regeneration of urothelial cells due to tumor or inflammation might release CEA into the urine [17]. It was suggested by some authors that elevated urinary CEA values, once infection is excluded, are specific to urothelial carcinoma, as the levels are normal in association with nonurothelial tumors such as hypernephroma, prostatic and colorectal carcinoma [16]. Even if the plasma levels are raised, the urinary levels rise only when such tumors involve the urinary tract by infiltration. Urinary CEA like activity was found to be increased in 61% of patients with transitional cell carcinoma of the bladder [3]. On the other hand, serial measurement of serum CEA was noted to judge response of advanced urothelial tumors to chemotherapy [18]. In this series, after exclusion of acute infection, urine CEA levels were raised in 86% of patients with bilharzial carcinoma and in 62% of patients with nonbilharzial carcinoma of the urinary bladder. There was also a significant difference between urine CEA in cancer patients and in the controls.

Morning samples of urine were shown to be more informative because of the benefit of overnight exposure of urine to the tumor [11]. On the other hand, 24 hour urinary CEA was advised to be measured, as it was shown to be more informative being elevated in 81% of patients with active tumors [7]. In this series, CEA was measured in the morning samples of urine only.

Urinary infection was found to spoil the use of urinary CEA as a diagnostic procedure [11]. The simple presence of bacteria in the urine irrespective of their identity or number has no influence on urinary CEA [19]. It is the inflammation of the urothelium that is responsible for the production and release of CEA. Therefore, only in the presence of symptoms and signs, and in the presence of pyuria, urinary tract infection is considered to exist. Infection was considered to be present if 5 leukocytes/HPF or more exist in the urine [17]. On the other hand, the effect of infection can largely be eliminated by routine use of midstream specimens of urine and its examination for pus cells and organisms [1]. However, in this series patients showing symptoms and signs of acute urinary tract infection were excluded from the study. Midstream samples of urine were collected and examined for pus cells.

Serum CEA levels were found by some investigators to be of little value in the diagnosis of transitional cell carcinoma [3]. In addition, there was no correlation between serum and urinary CEA values. On the other hand, serum and urine CEA were found by other investigators to have less than enough of the diagnostic accuracy required for clinical diagnosis of urothelial cancer [20]. In this series, there were many patients with raised urinary CEA levels while serum CEA levels were within normal, but in two cases only serum CEA levels were raised with normal urinary CEA levels.

With nonmetastatic bladder carcinoma, plasma CEA levels in one study were raised only in 42% of the patients, but with the development of extravesical metastatic spread, the incidence of raised plasma CEA values increased to 85% [6]. On the other hand, in a case report, CEA was elevated with the development of adenocarcinoma in the reconstructed bladder following ileocystoplasty [21]. Some authors also stressed the use of plasma CEA in assessing response to chemotherapy in advanced bladder cancer [22]. In this series, serum CEA levels were raised in 39% of patients with bilharzial carcinoma of the urinary bladder and in only 10.5% of control cases. This indicates that serum CEA is of little diagnostic value in carcinoma of the urinary bladder although there was a significant difference between the mean of serum CEA in patients with carcinoma and the controls. There were no documented cases with distant metastases but the patients with raised serum CEA levels in this series might have spread to the regional lymph nodes or have distant micrometastases elsewhere in the body.

Regarding the tumor stage, some authors have found a correlation between it and the level of CEA in urine, the higher the stage the higher the level [3,5,23,24] However, others found no correlation [7,10,11,19,25] In this series, a definite relationship was found between the stage of malignancy and the levels of urinary CEA in the urine; the higher the stage the higher the level of CEA. On the other hand, serum CEA was in another study found to increases with increasing extent of cancer [3]. On the contrary, no correlation was found in the present study [5,10,11]. In this series, no relationship could be detected between the stage of malignancy and the level of serum CEA.

Many authors found no correlation between the grade of malignancy and urine CEA levels [1,5-7,19,25,26]. However, others have advocated that the less differentiated the tumor, the higher is the level of CEA in the urine [3]. In this series, no correlation was found between the urinary CEA levels and the different grades of malignancy.

The levels of urinary CEA in this series in bilharzial carcinoma were higher than the levels in nonbilharzial carcinoma, though the difference was insignificant (P > 0.05 and < 0.10). This difference may be attributed to the associated chronic cystitis which is always present in bilharzial carcinoma as chronically stimulated urothelium forms CEA at an increased rate, and therefore increased release of CEA in the urine occurs [5,27]. It was also found by some authors that in premalignant bilharzial lesions, CEA in the urine reached levels as high as those encountered in frank bladder malignancy [8]. These premalignant lesions in association with bilharzial carcinoma may lead to this increase in CEA production. The other proposed cause for this difference is the tumor mass, as bilharzial carcinoma tends to be more bulky [15], and according to some authors, CEA in the urine increases with the increase in the size of the tumor [6,19,23,26]. This difference is not due to the histopathological difference between bilharzial and nonbilharzial carcinoma as no difference could be detected between urinary CEA in squamous cells carcinoma and transitional cell carcinoma in this series.

## Conclusion

Estimation of urine CEA can be useful in the early detection of carcinoma of the urinary bladder among high-risk people, particularly if provoked by bilharziasis. It adds more data to the staging of tumors, and hence to the prognosis after treatment.

## Competing Interests

The authors declare that they have no competing interest

## Authors' contributions

GMS: Chief author. created the idea, co-author selection, participated in the design of the study, performed clinical work and drafted the manuscript

WHEI-M: Carried out immunoassay, revised the manuscript draft

MSE: Participated in the clinical work, participated in the design of the study and performed the statistical analysis (cooperating with a specialist statistician)

NRD:Approved study design, participated in the sequence alignment, drafted the manuscript, revised references and revised final copy before delivery *[Correspondent]*

All authors read and approved the final manuscript
